# Supplementary material for: Asset-level assessment of climate physical risk matters for adaptation finance
Source: Nat Commun. 2024 Jul 1;15:5371. doi: 10.1038/s41467-024-48820-1 (PMC11217445; doi:10.1038/s41467-024-48820-1)
Supplement: Supplementary file 1 — Supplementary Information [file 41467_2024_48820_MOESM1_ESM.pdf]

Supplementary materials to Asset-level assessment of climate physical risk matters for adaptation finance

Giacomo Bressan[1] Anja Duranovic[2] Irene Monasterolo[2*]
Stefano Battiston[3,4]

[1]Institute for Ecological Economics, Vienna University of Economics and Business, Vienna, Austria
[2]Utrecht University School of Economics, Faculty of Law, Economics and Governance, Utrecht University, Utrecht, The Netherlands
[3]Department of Banking and Finance, University of Zurich, Zurich, Switzerland
[4]Research Institute for Complexity, Department of Economics, Ca' Foscari University of Venice, Venice, Italy
[*]i.monasterolo1@uu.nl

April 23, 2024

1 Additional information on the database model

1.1 Business lines data

To provide an assessment of physical risk that accounts for differences in firms' business lines composition, we collect data on firms' revenues by business lines.

This is a labour-intensive task which requires a large amount of information from firms' balance sheet and non-financial reporting, as well as data sourced from third party data providers. In addition, firms' reporting is not standardized (every firm uses its internal definitions of segments). In order to disaggregate the total revenues of firms by economic activity, we leverage information on business units, product types, and

their respective sales quantities and prices, and information on assets within the firms' portfolios. Our methodology combines information from firms' reports, websites, and other publicly available sources, together with other types of information (e.g., on mining commodities' prices from S&P[1], information on energy prices from Eurostat[2] and EIA[3], information about other commodities' prices from the World Bank[4], etc.).

The disaggregation of revenues to business lines can be scaled up using machine learning (ML) techniques, to avoid a labour-intensive collection process. This can be done with simple algorithms, such as classifier chains, or more complex deep learning nets. However, manual reclassification is inevitable to create the relevant training data. The role of manual classification for business lines data is described in Supplementary Figure 1, while the ML approach to the problem is described in Supplementary Figure 2.

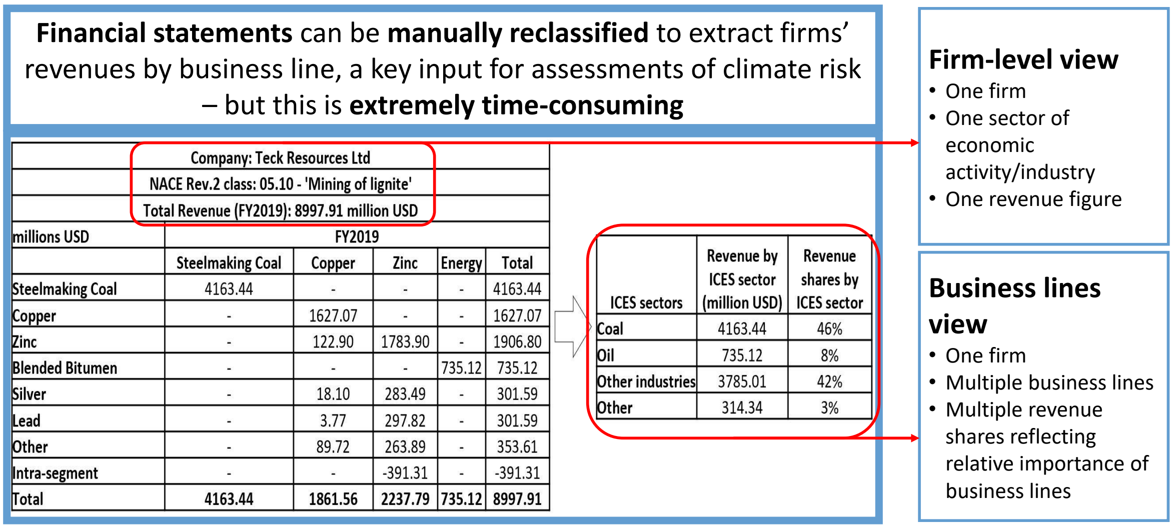

Company: Teck Resources Ltd					
NACE Rev.2 class: 05.10 - 'Mining of lignite'					
Total Revenue (FY2019): 8997.91 million USD					
millions USD	FY2019				
	Steelmaking Coal	Copper	Zinc	Energy	Total
Steelmaking Coal	4163.44	-	-	-	4163.44
Copper	-	1627.07	-	-	1627.07
Zinc	-	122.90	1783.90	-	1906.80
Blended Bitumen	-	-	-	735.12	735.12
Silver	-	18.10	283.49	-	301.59
Lead	-	3.77	297.82	-	301.59
Other	-	89.72	263.89	-	353.61
Intra-segment	-	-	-391.31	-	-391.31
Total	4163.44	1861.56	2237.79	735.12	8997.91

ICES sectors	Revenue by ICES sector (million USD)	Revenue shares by ICES sector
Coal	4163.44	46%
Oil	735.12	8%
Other industries	3785.01	42%
Other	314.34	3%

Supplementary Figure 1: A firm is considered as a portfolio of business lines and geographically distributed assets, rather than a monolithic entity. The information on total revenues is, thus, not enough for climate risk assessment, and the reclassification of firms' activities by business lines and product types is needed. The first necessary step is the manual reclassification of revenues based on a set of information from financial statements and other sources. While automation is possible, the heterogeneity of individual firms' reports and their complexity require the manual step for the creation of training data. Retrieved from: Teck Resources Ltd's Annual Report for fiscal year 2019 (FY2019; https://www.teck.com/media/2019-Annual-Report.pdf). Source: authors' own elaboration.

[1] https://www.spglobal.com/en/
[2] https://ec.europa.eu/eurostat/web/energy/data/database
[3] https://www.eia.gov/electricity/,https://www.eia.gov/naturalgas/
[4] https://www.worldbank.org/en/research/commodity-markets

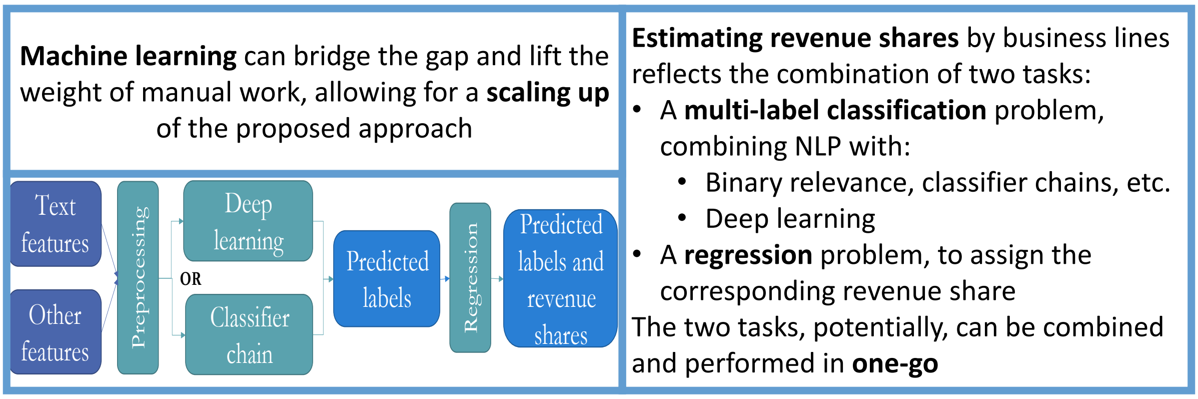

Supplementary Figure 2: Various Machine Learning (ML) techniques can bridge the gap and scale up the process of revenue collection by business line. However, only supervised machine learning algorithms are suitable for the problem and require a substantial amount of training data. That is why a manual reclassification step is inevitable. The multi-label classification problem can be tackled using algorithms such as classifier chains, or more complex deep learning approaches. The regression problem can be tackled either in one-go within a neural network, or separately using a dedicated regression algorithm or neural network.

1.2 Asset-level data

In a further downscaling step, we collect, clean, geolocalize, consolidate and match to the respective owners a database of physical assets exposures, which can be subsequently matched to geolocalized information on physical hazards. The information about physical assets of firms' is available from Refinitiv Eikon and Standard & Poor's.

For this study, we focus primarily on the energy sector. We extract information on LNG plants (from Refinitiv Eikon), mines, processing facilities and power plant units (from S&P). The choice is justified by the relevance of physical risk for the sector (see Schaeffer et al., 2012[1] for a comprehensive review). Moreover, since energy firms are considered as "sin stocks"[2], there is the need for a better understanding of the climate risks exposure of the sector, and the potential economic and financial implications of climate change. Our combined databases return 123,340 physical assets globally. 3,493 are located in Mexico and we reconstruct their chain of ownership. Ultimately, we link 1,820 physical assets to firms, both Mexican and internationally owned, that are invested in by European financial actors.

Note that asset-level data are, as of now, scattered and of limited quality. In fact, while interest in spatial finance is increasing, and disclosure and advances in artificial intelligence are progressively improving the amount and quality of available data, a complete dataset of physical assets from firms does not exist yet.

To overcome these limitations, we leverage multiple asset-level databases and implement an articulated preprocessing pipeline to estimate missing values related to fundamental asset characteristics such as value, production capacity or residual life. The purpose of the preprocessing pipeline is to clean the dataset from irrelevant entries (for example, assets permanently closed or projects abandoned) and to ensure that all variables relevant for the analysis (e.g. production capacity, coordinates of location, residual life and value) are available for each asset. We use relatively simple but established methodologies to impute capacities, residual lives and values where missing, such as Net Present Value (NPV) formulas, estimations by cost curves, regressions or regional averages by asset type or commodity. For example, we impute assets' capacity (i.e., any measure of production volumes of a given plant, such as electricity generated) using country or regional averages by commodity; residual lives by combining information on lifespans (sourced e.g., from Statista[5], McKinsey[6], International Financial Reporting Standards (IFRS)[7]) with assets' first production year; and latitude/longitude coordinates using the OpenCage API. For the NPV calculation, we use price information from e.g., historical data (sourced from Refinitiv Eikon), Statista, the US Energy Information Administration[8], or the Oxford Institute for Energy Studies.[9] As the database is global in scope, it is well-positioned for extensions of the analysis to other countries or sectors. Similarly, the preprocessing pipeline can be easily adapted to new asset information once available.

At the end of the process, the consolidated dataset contains information on geolocalized assets with an estimation of the respective production capacity, monetary value and useful life. It also contains information on technology, operating status and ownership.

Moreover, using asset values, we attribute the contribution of the individual asset to the business-line and revenue stream of the firm. This step allows us to downscale climate risk assessment to the fundamental business units of the firm, considering their potentially different exposure to climate-related hazards, due to geographical location.

[5] https://www.statista.com/
[6] https://www.mckinsey.com/
[7] https://www.ifrs.org/
[8] https://www.eia.gov/
[9] https://www.oxfordenergy.org/

1.3 Equity data

We extracted from the Ownership Universe in the Refinitiv Eikon Screener App[10] the dataset of exposures via equity contracts to publicly listed firms of European financial actors, as of June 2020.

To obtain equity exposures of European financial actors to publicly listed firms that have assets in Mexico, we consider all European financial actors headquartered in the European Union (EU)[11]. In addition, we consider financial actors from the European Free Trade Association (EFTA) Member States[12], European microstates[13], the United Kingdom (GB) (together with its Crown dependencies[14]), and all the Overseas Territories[15] of the countries mentioned. Whenever the data on the country of headquarters (HQ) of an investor is not available, we use the country of incorporation. Furthermore, we consolidate investors at the level of ultimate parent[16]. Finally, building on the approach in Battiston et al., 2017[3], we group investors consolidated into five investor types based on their NACE Rev.2 4-digit codes.

After the consolidation of individual firms' ownership, the consolidation on investor parent - investor level, and the retaining of ordinary shares and American Depositary Receipts (ADRs)[17], we obtain a dataset of 17,147 individual equity holdings[18] of 1,014

[10] https://eikon.thomsonreuters.com/index.html.

[11] Austria (AT), Belgium (BE), Bulgaria (BG), Croatia (HR), Cyprus (CY), Czechia (CZ), Denmark (DK), Estonia (EE), Finland (FI), France (FR), Germany (DE), Greece (GR), Hungary (HU), Ireland (IE), Italy (IT), Latvia (LV), Lithuania (LT), Luxembourg (LU), Malta (MT), Netherlands (NL), Poland (PL), Portugal (PT), Romania (RO), Slovakia (SK), Slovenia (SI), Spain (ES), and Sweden (SE).

[12] Iceland (IS), Liechtenstein (LI), Norway (NO), and Switzerland (CH).

[13] Andorra (AD), Monaco (MC), and San Marino (SM).

[14] Guernsey (GG), the Isle of Man (IM), and Jersey (JE).

[15] Anguilla (AI), Bermuda (BM), British Virgin Islands (VG), Cayman Islands (KY), Curacao (CW), Gibraltar (GI), and Greenland (GL).

[16] For consolidating investors, we apply the methodology used by the Refinitiv Eikon, and assign investor consolidated if the direct or indirect ownership interest exceeds 50%. However, whenever the investor consolidated is a sovereign, a foundation, or a corporate entity domiciled outside of European countries considered, the entity that is a corporate located in one of the countries that are considered, and that is closest to the investor's ultimate parent on the ownership pathway, is assigned as investor consolidated.

[17] We consider ordinary shares (including both fully- and partly-paid ordinary shares) and American depository receipts (ADR) because the investors' equity exposures to these two types of financial instruments accounts for almost 90% of the total value of equity exposures held by legal entities in the the dataset originally retrieved from Eikon, and because to both of them, a similar equity valuation approach can be applied.

[18] Characteristics of equity securities held by a given investor include: security identifiers - Reuters Instrument Code (RIC) and International Securities Identification Number (ISIN), type of security, filing date and filing type, and holdings value in USD.

European investors consolidated[19], in 177 firms[20] that have assets in Mexico, via 199 different equity instruments. The total exposure value of European investors amounts to 290.11 billion USD (as of 30 June 2020). For each investor consolidated type, Supplementary Table 1 reports the number of individual equity holdings and the holdings value (in billion USD). The investors consolidated with the largest equity exposures are Norges Bank[21] (33.59 billion USD), Legal & General Group PLC[22] (11.54 billion USD), UBS Group AG[23] (8.39 billion USD), and Deutsche Bank AG (7.99 billion USD)[24]. The equity issuers are headquartered in 25 different countries - the majority of which in Canada (60), US (31), Mexico (23), and Japan (11).

Investor consolidated type	NACE Rev.2 4-digit codes	Number of investors consolidated with a given type	Percentage of investors consolidated with a given type	Number of individual equity holdings by investor type in firms that have assets in MX	Value of equity holdings by investor type (in billion USD) in firms that have assets in MX
Banks	K64.1	153	15.09 %	5,568	94.81
Insurance and pension funds (IPFs)	K65.1-K65.3, K66.2	62	6.11 %	2,015	22.68
Investment funds (IFs)	K64.2, K64.3, K64.9.1, K66.1.2, K66.3	620	61.14 %	6,863	144.80
Other credit institutions (OCIs)	K64.9.2, K64.9.9	74	7.30 %	1,883	20.76
Other financial services (OFSs)	K66.1.1, K.66.1.9	105	10.36 %	818	7.06

Supplementary Table 1: Classification of investors consolidated into types based on their NACE Rev.2 4-digit codes. For each investor type, the number and relative share of investors consolidated are first summarized. Then, the number of individual holdings in firms with assets in Mexico and their value (in billion USD) are presented. Retrieved from: Refinitiv Eikon. Source: adapted from Battiston et al., 2017[3]; own calculations.

[19]Investors' characteristics include: investor name, Refinitiv PermID, Legal Entity Identifier (LEI), country of HQ, country of incorporation, NACE Rev.2 class (i.e., 4-digit code), and NACE Rev.2 section; and investor consolidated name, Refinitiv PermID, Legal Entity Identifier (LEI), country of HQ, country of incorporation, NACE Rev.2 class (i.e., 4-digit code), and NACE Rev.2 section.

[20]Characteristics of equity securities' issuers include: firm name, Refinitiv PermID, Legal Entity Identifier (LEI), country of HQ, NACE Rev.2 class (i.e., 4-digit code), and NACE Rev.2 section.

[21]Via its subsidiary Norges Bank Investment Management (headquartered in NO)

[22]Via its subsidiary Legal & General Investment Management Ltd (headquarted in GB)

[23]Via its subsidiaries UBS Asset Management Switzerland AG, UBS AG, UBS Switzerland AG, UBS Swiss Financial Advisers AG (all headquartered in CH), UBS Asset Management UK Ltd, UBS Reorganisation 2011-02 Ltd (both headquartered in GB), UBS Europe SE, UBS Asset Management Deutschland GmbH (both headquartered in DE), and UBS Gestion SGIIC SA (headquartered in ES).

[24]Via its subsidiaries DWS Investment GmbH, DWS International GmbH (both headquartered in DE), DWS Investments UK Ltd (headquartered in GB), Deutsche Bank Luxembourg SA (headquartered in LU), Deutsche Bank Suisse SA, DWS CH AG (both headquartered in CH), and Deutsche Wealth Management SGIIC SA (headquartered in ES).

For each equity instrument, we gather information on dividends per share (DPS) and earnings per share (EPS)[25] For 67.34% of instruments, DPS data is available for at least one period.

2 ICES sectors

We use the ICES model[4,5] to compute macro-economic shocks on business lines conditioned to different climate scenarios. The description of ICES sectors is in Supplementary Table 2 below.

ICES sector	Description
Rice	Rice: seed, paddy (not husked).
Wheat	Wheat: seed, other.
Other grains	Other Grains: maize (corn), sorghum, barley, rye, oats, millets, other cereals.
Vegetables and Fruits	Veg & Fruit: vegetables, fruit and nuts, edible roots and tubers, pulses.
Oil seeds	Oil Seeds: oil seeds and oleaginous fruit.
Cane & Beet	Cane & Beet: sugar crops.
Fibres crops	Fibres crops.
Other crops	Other crops e.g. stimulants, spice and aromatic crops, forage products, other raw vegetable materials nec.
Other Agr-Frs-Fsh	Other agricultural, forestry and fishery products e.g. cattle, other animal products, forestry, fishing.
Coal	Coal: mining and agglomeration of hard coal, lignite and peat.
Oil	Oil: extraction of crude petroleum, service activities incidental to oil and gas extraction excluding surveying.
Gas	Gas: extraction of natural gas, service activities incidental to oil and gas extraction excluding surveying; Gas manufacture, distribution.

[25]Equity prices and the firms' Earnings Per Share (EPS) and Dividend Per Share (DPS) are obtained from Standard & Poor's (https://www.spglobal.com/en/).

Other industries	Other mining extraction, lumber, paper & paper products, manufacture of pharmaceuticals, rubber and plastic products, fabricates metal products, motor vehicles, other transport equipment.
Food industries	Food products e.g. cattle meat, other meat, vegetable oils, dairy products, beverages and tobacco products, other food product nec.
Light industries	Manufacture of textiles, apparel, leather and leather products, computer, electronic and optical products, electrical equipment, machinery and equipment nec.
Oil products	Petroleum & Coke: manufacture of coke and refined petroleum products.
Chemicals	Manufacture of chemicals and chemical products.
Non Mineral Products	Manufacture of other non-metallic mineral products.
Iron & Steel	Iron & Steel: basic production and casting.
Non-Ferrous metals	Non-Ferrous Metals: production and casting of copper, aluminium, zinc, lead, gold, and silver.
Electricity-Fossil	Fossil electricity; steam and air conditioning supply.
Electricity-Nuclear	Nuclear electricity; steam and air conditioning supply.
Electricity-Hydro	Hydropower electricity; steam and air conditioning supply.
Electricity-Renewable	Renewable electricity; steam and air conditioning supply.
Services	Services e.g. water supply, wholesale and retail trade, accommodation, recreation, other government services, education, human health.
Construction	Construction: building houses factories offices and roads.
Other transport	Land transport and transport via pipelines.
Water transport	Water transport.
Air Transport	Air transport.

Supplementary Table 2: This table reports the sectoral disaggregation used in the Intertemporal Computable Equilibrium System (ICES) model, with the corresponding descriptions. nec stands for not elsewhere classified. Source: Bosello et al., 2020[6], Delpiazzo et al., 2021[7].

3 Probabilistic risk assessment

The procedure to perform the probabilistic risk assessment on assets is described in Supplementary Figure 3. Supplementary Figure 4 shows all historical hurricane tracks in our sample by category.

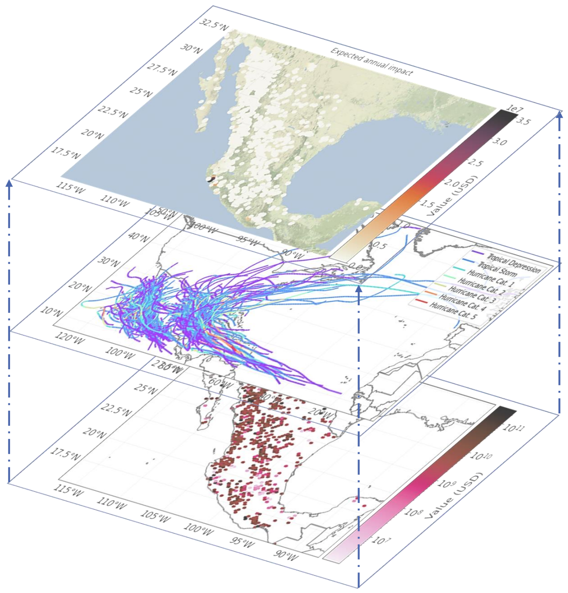

Impact

- Direct damages computed at different return periods and on average
- Info feeds into equity shocks and valuation adjustments

Hazards

- Tropical cyclones as computed in the CLIMADA model
- Other acute hazards shall be considered in further studies

Geolocalized assets

- Referenced by latitude/longitude
- Defined by asset type (e.g. power plant, mine, etc.)
- Non-financial variables (e.g. capacity, residual life)
- Financial variables (e.g. value)

Supplementary Figure 3: Workflow of our approach to probabilistic disaster risk assessment for tropical cyclones in Mexico, covering: (bottom panel) spatially explicit exposures, i.e. assets' geographical distribution and assets' characteristics. (Mid panel) historical (and synthetic, not shown) hurricane tracks as obtained from CLIMADA. (Top panel) expected annual impacts on physical assets. The workflow proceeds from the bottom to top of the figure. Source: authors' elaboration on CLIMADA (https://github.com/CLIMADA-project, [8], [9]) outputs.

4 Asset-level damages with a finer grid

To complement our analysis, we investigate the effect of using a finer spatial grid (150 arcsec, or approximately 4.5 km at the Equator, instead of 0.2 degrees, or approximately 22 km at the Equator) on the asset-level damages. To do so, we use CLIMADA's data API[26] to source pre-computed, 150 arcsec gridded tropical cyclones maximum intensities over Mexico. The API data are simulated using 10 synthetic tracks for each real track, historical data for years 1980-2020, reference year for climate conditions 2040, and a wind

[26] https://climada-python.readthedocs.io/en/latest/tutorial/climada_util_api_client.html

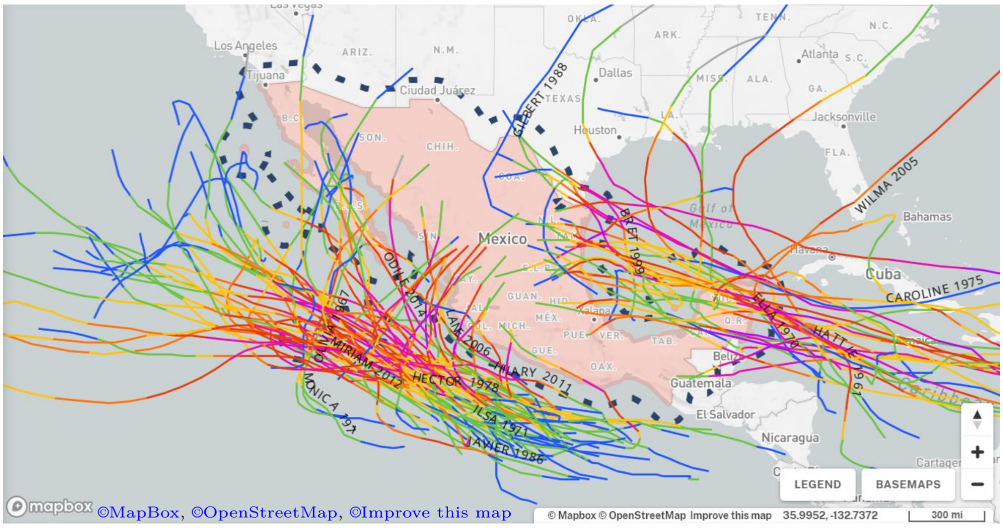

Supplementary Figure 4: Historical hurricane tracks within 60 nautical miles of Mexico, between 1880 and 2022. Only major hurricanes (categories 3, 4, and 5) are shown. Legend by track colour: purple, category 5; violet, category 4; red, category 3; orange, category 2; yellow, category 1; green, tropical storm; blue, tropical depression; grey, extratropical storm. Colours associated to hurricanes of categories less than 3 may appear on the map as major hurricanes lose strength. Source: authors' elaboration on NOAA, historical hurricane tracks data (https://coast.noaa.gov/hurricanes), underlying map from Mapbox (https://www.mapbox.com/about/maps, https://labs.mapbox.com/contribute/), and OpenStreetMap (https://www.openstreetmap.org/about/).

speed interpolation step of one hour. Hence, there are four key differences between our set-up and the one used in the API: the grid spacing, the number of simulated tracks, the interpolation step, and the reference period for historical data. We use 2040 as a reference year for the comparison since it is the only common year between our set of years (2035, 2040, 2045, 2050) and the API's set of years (2040, 2060, 2080). For both the API data and our original assessment (see Methods), we compute asset-level damages as a percentage of asset value.

We compare the two cases and find that the difference between the finer and coarser grid is negligible (<0.007%) in the case of EAI. When considering RP250, we find a small effect in the adjustment of the damages (slight increase or decrease). The maximum of the average difference in damages across all assets using the finer grid with respect to the coarser grid is 0.38% (i.e. 0.44% with coarser grid vs 0.82% with finer grid). The average difference does not vary largely by asset type, ranging from a minimum of 0.07% for power plants to a maximum of 1.09% for liquefaction plants.

Importantly, for the majority of assets, differences in damages using the finer and coarser grid are negligible, with the 75th percentile of the distribution of differences

Statistic	4026_eai	4045_eai	4060_eai	4026_rp250	4045_rp250	4060_rp250
mean	0.00	0.01	0.01	0.23	0.38	0.34
std	0.02	0.02	0.02	1.21	1.52	1.37
min	-0.04	-0.05	-0.04	-4.14	-3.99	-3.57
25%	0.00	0.00	0.00	0.00	0.00	0.00
50%	0.00	0.00	0.00	0.01	0.02	0.01
75%	0.00	0.00	0.00	0.15	0.22	0.19
max	0.19	0.24	0.21	11.48	14.38	12.80

Supplementary Table 3: Summary statistics for the difference in asset-level damages obtained comparing the 0.2 degrees grid used in this study with the 150 arcsec grid sourced from CLIMADA's API. Four key differences exist between our set-up and the one used in the API: the grid spacing, the number of simulated tracks, the interpolation step, and the historical data timeframe. Summary statistics are presented by row, in the following order: mean (mean), standard deviation (std), minimum (min), 25th percentile (25%), 50th percentile (50%), 75th percentile (75%), and maximum (max). Each column represents a measure of damages (Expected Annual Impacts (EAI) or 250-years Return Period (RP250)) for a different scenario, for the reference year 2040, in the following order: EAI, year 2040, Representative Concentration Pathway 2.6 (RCP2.6, 4026_eai); EAI year 2040, RCP4.5 (4045_eai); EAI, year 2040, RCP6.0 (4060_eai); RP250, year 2040, RCP2.6 (4026_rp250), RP250, year 2040, RCP4.5 (4045_rp250); RP250, year 2040, RCP6.0 (4060_rp250). Values in the cells are expressed in percentages. Source: authors.

being 0.2%. In a few cases, some large changes occur, with maximum higher damage from the finer grid being 14% and minimum lower damage from the finer grid being -4%. However, only 0.66% of assets have a higher damage from the finer grid that exceeds 10% in the RP250 case. Thus, we can conclude that using the finer grid leads to some assets suffering more damages in our framework. At the firm level, results are less clear, as some netting may occur (i.e. some firm's assets may be more damaged, but others may be less damaged, with the two effects reducing firm-level changes). Still, on average, firm- and portfolio-level damages can be expected to marginally increase. Importantly, we tested the refined grid also in the proxy case finding that no change in damages occurred, neither for EAI nor for RP250. Thus, our main results on underestimation are not impacted by the usage of a coarser grid.

Full statistics for the comparison are reported in Supplementary Table 3 below.

5 Scenario combinations

A summary of the scenario combinations used in the model is provided in Supplementary Table 4.

Model	SSPs	RCPs	Scenario Combinations	Year
CLIMADA	Not used	Used	RCP 2.6, RCP 4.5, RCP 6.0	2035, 2040, 2045, 2050
ICES	Used	Used	SSP2-RCP6.0, SSP3-RCP2.6, SSP3-RCP4.5, SSP5-RCP4.5	2035, 2040, 2045, 2050
CDDM	Used	Used	SSP2-RCP6.0, SSP3-RCP2.6, SSP3-RCP4.5, SSP5-RCP4.5	2035, 2040, 2045, 2050

Supplementary Table 4: Summary of the scenario framework in the model used for hazards (CLIMADA), in the macroeconomic model (Intertemporal Computable Equilibrium System; ICES), and in the Climate Dividend Discount Model (CDDM) presented in this paper. Scenarios are distinguished between Shared Socioeconomic Pathways (SSPs; not used/used) and Representative Concentration Pathways (RCPs, not used/used) and their combinations are presented for each model. All models use the same time steps.

6 Rationale for the three-stages model

The economic rationale behind the separation in a three-stages model has been first introduced by Sharpe et al., 1999[10]. The main idea is to separate the growth trajectory of a firm in three main phases: first, a growth phase, where firms typically enjoy rapidly expanding markets, high profit margins and an abnormally high rate in earning per share (s.c. supernormal growth). Such a strong expansion is generally not sustainable and hence firms move into a second phase, called transition phase, where earnings growth slows as competitive pressure erodes margins or as sales decline because of market saturation. In this phase, earnings' growth can still be above average but starts to decline towards the growth rate of the economy. Ultimately, the firm moves into the third phase, or mature phase, where it reaches an equilibrium and earnings' growth reaches a level that can be sustained in the long term.

Clearly, firms can break this path and restart an abnormal growth phase, for example by entering new business lines or because of new market opportunities or technological advances. It is also important to consider that different firms are at different stages of such a cycle and that the stages can have different length for different firms. For example, some firms may benefit from extremely high growth rates for an extended period of time, while other will transition to maturity in a very short one.

Despite this heterogeneity, the three stage assumption represents a meaningful approximation and we apply it to all firms in our study, i.e. firms are not distinguished for

the growth phase they are in[27].

7 Sensitivity analysis

Models such as the CDDM have two main sensitivities: on the one hand, to the discount rate r and, on the other, to the initial long-term growth rate g_L. A higher r will imply lower equity valuations and hence, ceteris paribus, lower equity losses. By contrast, a smaller r will lead to larger losses due to the increased relevance of the terminal value component and intermediate dividends. g_L plays an opposite role. Reducing g_L reduces the magnitude of losses, while increasing it amplifies the magnitude. Importantly, one should notice that the difference $r - g_L$ is also relevant in itself, since as g_L approaches r losses are drastically amplified. To the limit, i.e. $r = g_L$, valuations go to infinity, while $g_L > r$ lacks an economic meaning in the DDM framework. Lastly, it should be noted that the sensitivity to g_L is stronger than the sensitivity to r, as illustrated empirically below in Supplementary Figure 5. This is stemming from the fact that losses originate from an adjustment in g_L, while r is kept constant.

In our results, we test the sensitivity of the model to these parameters by computing the CDDM losses on a $r_{range} \times g_{L,range}$ grid. For this application, we let r vary between 6% and 9%, at 1% steps, and g_L vary between 4% and 6%, at 1% steps. The case $r = g_L = 6\%$ is not computed as it would lead to infinite valuations.

8 Bootstrap results

To derive confidence intervals for the average portfolio loss and its Value at Risk (VaR), we use a bootstrap procedure. We apply the bias corrected and accelerated percentile method[11] over 15,000 samples to generate alternative portfolios. For each portfolio, we compute the mean of portfolio losses (using EAI) or its VaR (using RP250). In Supplementary Figure 6 we present the distribution of losses under four different cases, both excluding (panels (a) and (b)) and including (panels (c) and (d)) chronic risks.

[27] This would ultimately require multiple models for the different growth phases and further methodological developments to identify the phases, hence it is left for future work.

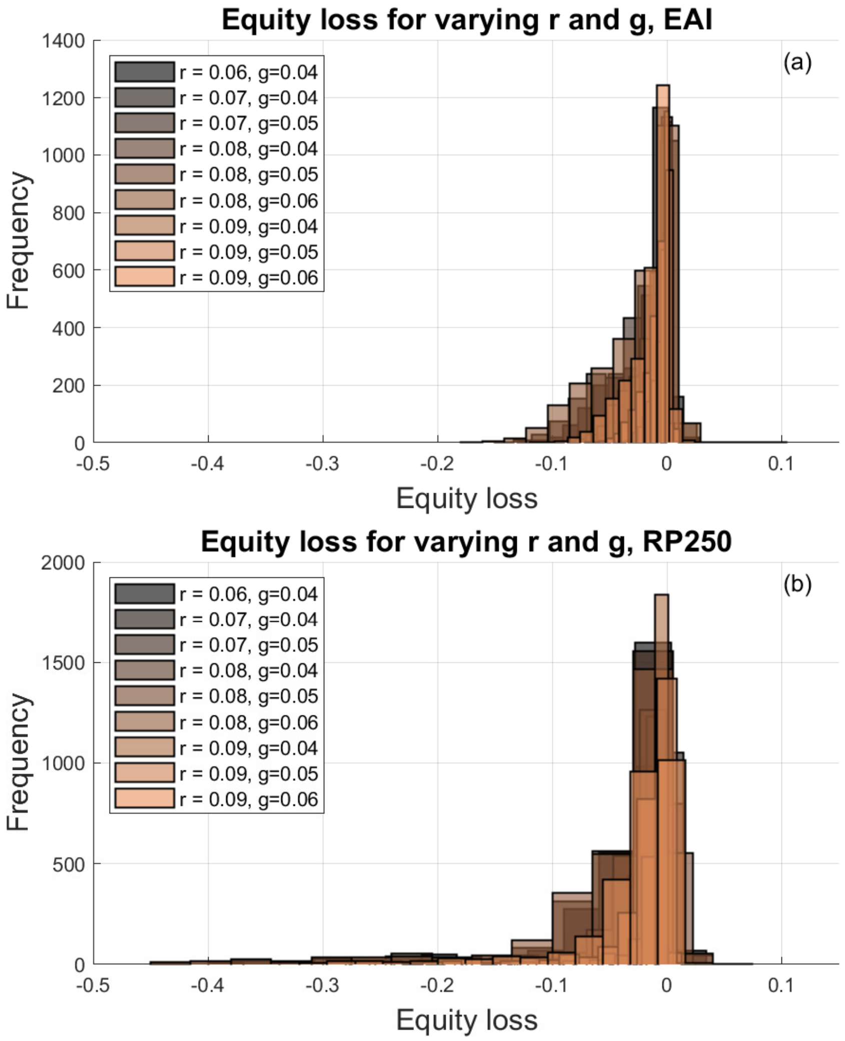

Supplementary Figure 5: Equity loss distributions, all scenario combinations, conditioned to Expected Annual Impacts (EAI - panel (a)) and conditioned to 250 Return Period impacts (RP250 - panel (b)) for tropical cyclones, for different values of r and g_L. Macroeconomic impacts from Intertemporal Computable Equilibrium System model; (ICES) are also included. Horizontal axis: loss defined as $\frac{\tilde{V}_0 - V_0}{V_0}$, where V_0 is the equity value without the shock and $\tilde{V}_0$ is the shocked equity value. Vertical axis: absolute frequency. For each scenario combination (i.e. each histogram) the distribution is taken across all time periods (2035, 2040, 2045, 2050).

9 Underestimation of losses from proxy data by scenario

Importantly, the underestimation of equity losses from using proxy data worsens as the RCP and year used in the equity valuation increase, as shown in Supplementary Figure 7. In fact, firms in the upper rows of the chart (higher-end RCP scenarios and later years) are in further negative territory, i.e. have worse underestimations, than firms in the lower

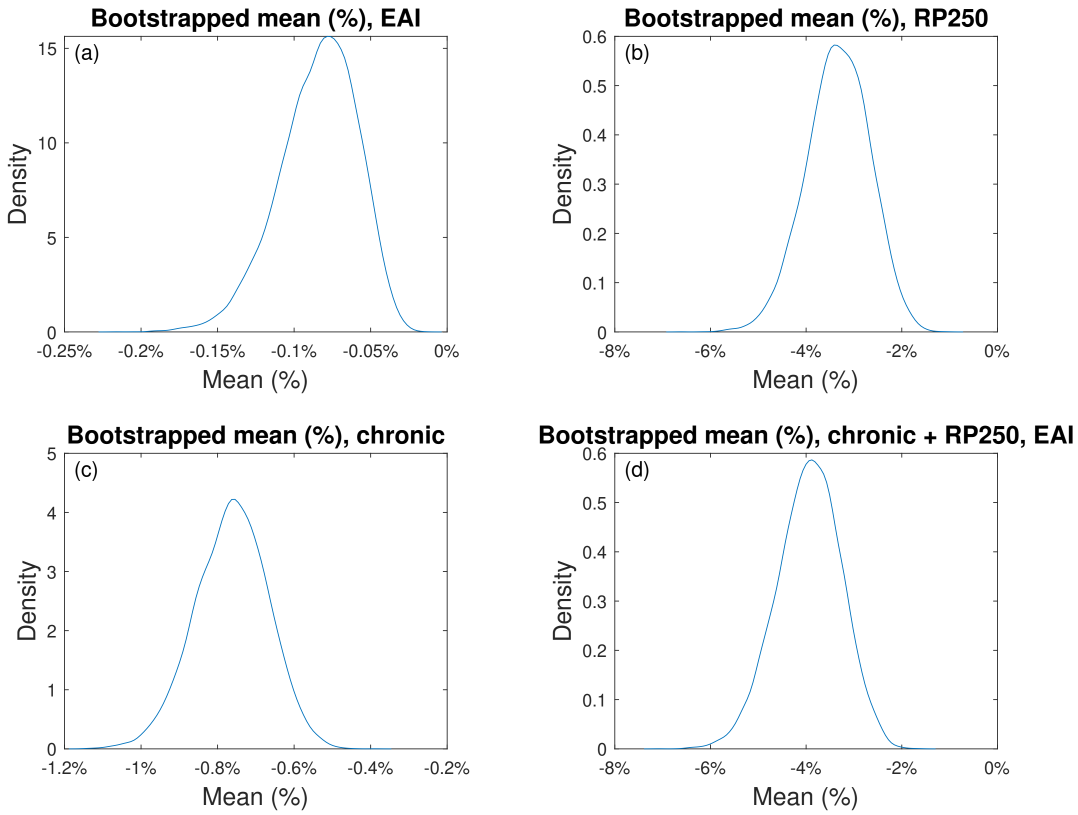

Supplementary Figure 6: Distribution of bootstrapped mean (in %) for four physical risk cases. To compute the bootstrap samples, we use the bias corrected and accelerated percentile method over 15,000 samples. Horizontal axis: mean (in %), i.e. the bootstrapped values for the average portfolio loss. Vertical axis: density. Panel (a): acute risks only, Expected Annual Impacts (EAI). Panel (b): acute risks only, 250-years Return Period (RP250) i.e. portfolio Value at Risk (VaR). Panel (c): chronic risks only. Panel (d): chronic and acute risks computed using RP250, i.e. portfolio VaR considering both chronic and acute risks.

rows (lower-end scenarios and earlier years).

10 Sensitivity of equity losses to adaptation measures

Here, we want to provide a first estimation of the errors on financial losses stemming from neglecting adaptation efforts in our model. To do so, we work on the derivative of Equation 11 in Methods (firm-level loss) with respect to the damages to assets, under a simplified setting.

We proceed as follows. First, we substitute Equations 7 and 10 in Equation 11, to describe the loss as a function of V and $\tilde{V}$. We then substitute $\tilde{g}$ and simplify it by setting

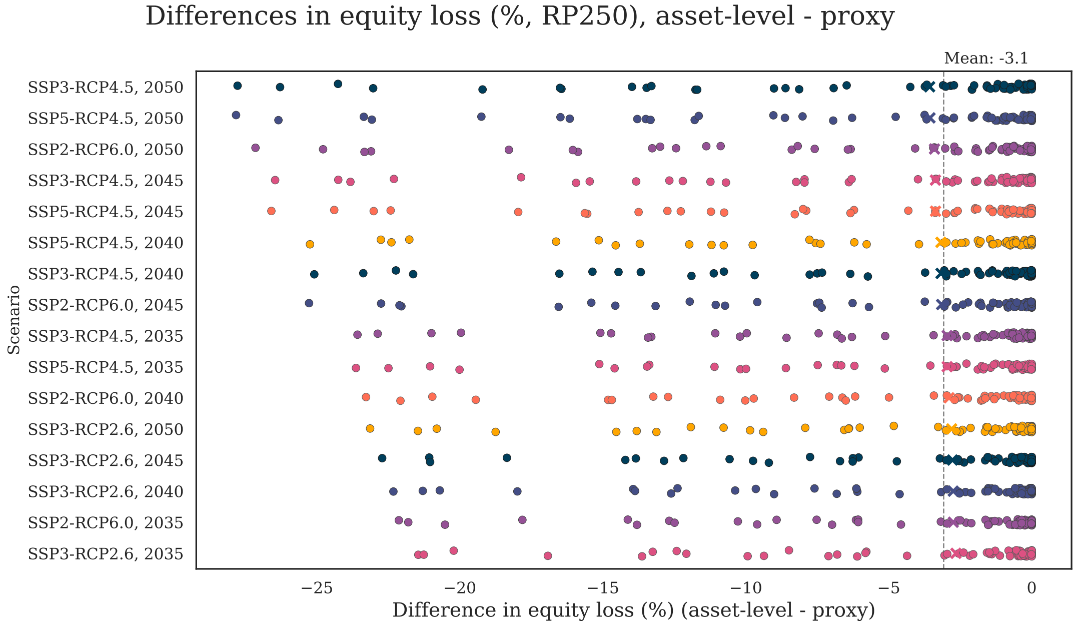

Supplementary Figure 7: Differences in losses on firms' equity computed using asset-level vs proxy data, all scenarios and years, 86 firms with available asset-level data. Vertical axis: scenario combinations described as "Shared Socioeconomic Pathway - Representative Concentration Pathway (SSP-RCP), year". Horizontal axis: difference in loss, expressed as "loss (asset-level) - loss (proxy)". A negative value indicates a loss and a positive value a gain. The larger the loss, the more negative (i.e. smaller) the value. Each dot represents a firm. A difference smaller (resp. larger) than zero represents a case where the loss is lower (resp. larger) using asset-level data, i.e. a case where the proxy data underestimates (resp. overestimates) the risk for a given firm. The dotted vertical bar represents the average difference in the dataset, at the value of -3.1%. The crosses represent the average difference for a given scenario.

$\frac{O_i}{O_b} = 1$, $K_j = 1$, $s_i = 1$. We set the first two terms of Equations 7 and 10 (see Methods) equal to A, as they are not a function of asset-level damages (thus, adaptation). We also consider that $\tilde{g} = 1/\delta$, to include the damage terms directly in the simplified equation. Here, δ is the same parameter as in Equation 9 of the main text, where we dropped the subscript j since we focus on a generic firm with one business line. δ is an increasing function of the relative damage. Thus we obtain:

$$\psi_j = \frac{\tilde{V}_{0,j,I} - V_{0,j}}{V_{0,j}} = \frac{\frac{D(1+g/\delta)}{(1+r)^t(r-g/\delta)} - \frac{D(1+g)}{(1+r)^t(r-g)}}{A + \frac{D(1+g)}{(1+r)^t(r-g)}} \tag{1}$$

We are now interested in computing the partial derivative of Equation 1 with respect to δ. In analytical form, this is not informative. Thus, we set values for the other variables in order to reduce the problem to analyzing a function of δ only. We set the parameters as follows: $r = 0.09$, $g = 0.06$, $t = 15$, $D = 1$, $A = 1$. Note that this is just one of the possible parametrizations, thus the following results are only an informed approximation

based on this parametrization. Most importantly, D and A will vary on a firm-by-firm basis, hence the actual sensitivity to damages will vary by firm.

In Supplementary Figure 8, we plot the function $\psi_j(\delta)$ for $\delta \in [1, 3]$ (since $\delta > 1$ holds by construction, see Methods Equation 9), and its derivative (also for $\delta \in [1, 3]$).

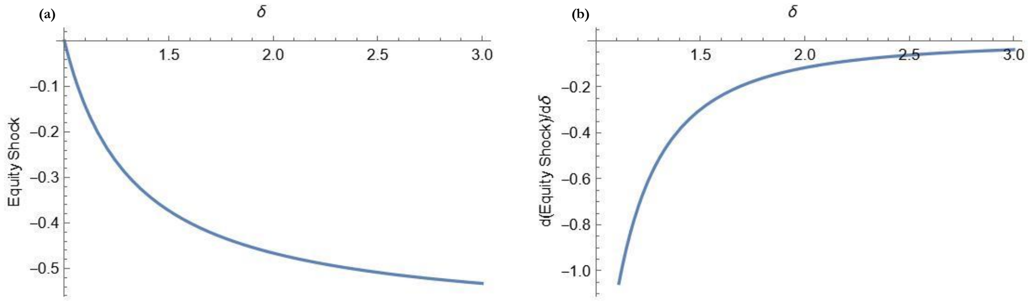

Supplementary Figure 8: Panel (a): plot of Equation 1 (i.e., the equity shock) as a function of δ, after fixed parametrization setting $r = 0.09$, $g = 0.06$, $t = 15$, $D = 1$, $A = 1$. X-axis: δ. Y-axis: equity shock. As δ increases, the equity shock becomes more negative (i.e., it is a higher shock in absolute value): higher damages lead to higher equity losses. Panel (b): plot of the derivative of Equation 1 (i.e., the slope of the variation of the equity shock) as a function of δ, after fixed parametrization setting $r = 0.09$, $g = 0.06$, $t = 15$, $D = 1$, $A = 1$. X-axis: δ. Y-axis: partial derivative of the equity shock with respect to δ. As δ increases, the slope of the variation of the equity shock becomes smaller: if damages are high, marginal adaptation efforts contribute little to reduce equity shocks.

From Supplementary Figure 8, we observe that the equity shock becomes more negative, implying larger losses, as damages increase. Note that the function in panel (a) of Supplementary Figure 8 is decreasing in δ. This is because the equity shock is expressed as a negative number, thus decreases (i.e., becomes more negative) for higher damages. This is intuitive, since more damages lead to larger asset-level losses and consequently larger equity losses. However, the derivative is increasing in our model, conditioned to the parametrization discussed above. This implies that marginal adaptation efforts to reduce very high damages have limited effect on equity losses. This enables us to obtain an approximation of the impact of adaptation in our model. For example, a reduction in δ from 2.5 to 2.0 has a relatively small impact on the equity loss, reducing it by only 0.04[28]. On the contrary, a reduction in δ from 2.0 to 1.5 has a much larger impact on the loss, reducing it by 0.09.

Mexico is characterized by high physical risks and at the same time by very little insurance penetration and highly inadequate adaptation measures. For instance, insurance penetration in Mexico has been estimated as about 7%.[29] Combining this information

[28] Note that in this sensitivity study we did not cap δ at 2.

[29] https://www.elasegurador.com.mx/blog/menos-del-7-de-los-inmuebles-en-mexico-estan-asegurados-contra-danos

with the result of the sensitivity analysis leads us to the conclusion that, as an approximation conditioned to our assumptions, current adaptation efforts would have very limited impact on our estimates on company-level losses. In other words, for the case of Mexico, δ is high (close to 2) and adaptation is small (i.e., corresponding to a variation of δ smaller than 7%). As we can see from the right side of panel (a) in Supplementary Figure 8 the reduction in damages from marginal adaptation efforts leads only to a limited reduction in equity losses[30]. Portfolio-level losses, and the results on underestimation, would also have a comparably low impact.

11 Uncertainty quantification

A full uncertainty analysis is out of scope of this paper. Still, it is possible to provide a quantification of the order of magnitude for the uncertainty associated with our estimates. We do so by combining the scenario analysis in our study with recent results about tropical cyclones uncertainty present in the literature.

We estimate the relative contribution of various sources of uncertainty to the total variation of the portfolio loss. Our aim is to understand how the portfolio loss in our model varies as a function of the inputs (e.g., wind speed, asset locations, etc.) and their uncertainty. Mathematically, we want to estimate the expression:

$$\Delta Loss_m = \sum_{i=1}^{N} \frac{\delta Loss_m}{\delta x_i} \Delta x_i \tag{2}$$

where the differential $\Delta Loss_m$ represents the variability in the portfolio loss in our model (either EAI or VaR), Δx_i the variability of the variable x_i (e.g., wind speed), and N is the number of variables. We proceed as follows. First, we observe that based on our results across scenarios, and focusing only on the variability arising from the wind speed, we have:

$$\Delta Loss_m = \frac{\delta Loss_m}{\delta x_{ws}} \Delta x_{ws} \tag{3}$$

where $Loss_m$ is the portfolio loss in our model (either EAI or VaR), and x_{ws} is the wind

[30] From Equation 9 in the main text, $\delta = 1 + \eta(L/V)$, where L is the loss on the firms assets. It is easy to verify that $\Delta\delta \leq \Delta(L/V)$

speed input, which varies across scenarios[12]. Then, we observe that from the literature[13] we can estimate:

$$\Delta Impact_c = \sum_{i=1}^{N} \frac{\delta Impact_c}{\delta x_i} \Delta x_i \tag{4}$$

Where $Impact_c$ represents the damages to physical capital derived by the authors of Meiler et al. (2023)[13], and x_i are the input parameters (e.g., damage function, scenario, etc.). Importantly, $Loss$ and $Impact$ are two different variables: the former is in fact a function of the latter, defined by the financial valuation. However, for the purpose of this analysis we can assume $\Delta Loss \leq \Delta Impact_m$, and specifically $\Delta Loss \sim \Delta Impact_m$, where the subscript m denotes the calculation in our model.

Furthermore, we know that the cross-scenario variability considered in our study is induced by the variation in wind speed. In Meiler et al., (2023)[13] the cross-scenario variability is induced by the variation in a wider parameter space, which includes the wind speed. Thus, we can write:

$$\frac{\Delta Impact_c}{\Delta Impact_m} \sim \frac{\Delta Impact_c}{\Delta Loss_m} = c \tag{5}$$

Where c is the ratio of the variability of impacts in Meiler et al., (2023)[13], including a larger set of inputs, to the variability of portfolio losses in our model. Consequently, we can estimate the total impact in our model, considering the wider set of variables, denoted as $\Delta Impact'_m$ by scaling up by the factor c the impacts we compute from considering only wind speed, as follows:

$$\Delta Impact'_m = c\, \Delta Impact_m \tag{6}$$

And consequently:

$$\Delta Loss'_m \sim \Delta Impact'_m = c\, \Delta x_{ws} \frac{\delta Loss}{\delta x_{ws}}. \tag{7}$$

Where $\Delta Loss'$ and $\Delta Impact'_m$ are the portfolio loss and the impacts (in our model) considering the whole set of inputs' uncertainties.

We now proceed to estimate the terms in the equations above. We first focus on Supplementary Equation 3. We observe that, in our study, we compute portfolio-level losses conditioned to different scenarios. These are defined by combinations of RCPs (namely RCP 2.6, 4.5, and 6.0) and years (namely 2035, 2040, 2045, and 2050). The results by scenario are presented in Supplementary Table 5. In the field of climate impact it is known that, due to the influence of natural variability, it is not possible to statistically distinguish across scenarios for tropical cyclones in specific years, e.g. 2035 compared to 2050. However, the mechanics of the CLIMADA model enables us to define an uncertainty range for the main input variable, namely the wind speed. In fact, in CLIMADA, the alteration in wind speed stemming from climate change is obtained through interpolation between present and future climates[8,12]. As a consequence, our scenario set-up describes an uncertainty range on the wind speed. Specifically, the scaling factor for the changes in intensity of tropical cyclones varies between 0.36 (for SSP2.6, year 2035) to 0.75 (for SSP4.5, year 2050). In other words, there is a range of 0.75-0.36=0.39 (or 107.79%) in the scaling factor for wind speed used in the tropical cyclone model. Note that the implementation in CLIMADA also alters tropical cyclones' frequencies. However, the changes in frequency are not significant for the North Atlantic basin[12]. This enables us to isolate the effect of the change in wind speed.

In our results (see Supplementary Table 5), the portfolio average (EAI) loss varies between -0.17% and -1.1%, and the VaR (RP250) varies between -4% and -3.2%. These represent a range of 0.93% (or, in relative terms, 547.06%) for the average, and 0.79% (or, in relative terms, 24.99%) for the VaR. Importantly, when we compute our results across scenarios, parameters other than the year and RCP (e.g., dividends, asset locations and values, etc.) are held constant. Thus, cross-scenarios changes are induced by changes in tropical cyclones, i.e., wind speed, only. Hence, the partial derivative of the valuation function with respect to the changes in the wind speed scaling factor is roughly equal to 24.99%/107.79% $\sim$ 0.25/1.08=0.23 (for RP250) and 547.06%/107.79% $\sim$ 5.47/1.08=5.06 (for EAI). These are computed as the ratios of the changes between the percentage variation in the wind speed scaling factors and the (relative) percentage variations in portfolio losses.

We now move to Supplementary Equation 4. In fact, the previous result covers only changes in wind speed. However, additional climate and non-climate related parameters could be altered in the analysis to provide a full uncertainty range. These include for example Global Circulation Models (GCM), wind-speed models, and socio-economic parameters such as SSPs or the distribution of assets. A complete analysis is beyond

the scope of our paper. However (Meiler et al., 2023)[13] recently tackled this problem in the context of tropical cyclones, using CLIMADA. In the study, the authors quantify the variation in damages to built assets for EAI and an RP100 event as a function of a large input uncertainty grid. This differs from our set-up, where we are interested in portfolio-level losses. However, results from (Meiler et al., 2023)[13] can be used to extrapolate a more general uncertainty range for our results. The authors first distinguish between climate and non-climate parameters for the model. Then, they define an input grid for all parameters, setting the uncertainty ranges of inputs. Finally, they compute the variation in damages to built assets (measured by EAI and RP100) with respect to a historical baseline, by running the model for all parameters' combinations in the grid. They show the following. Varying only climate-related parameters, changes in damages to built assets vary between -0.2% and 23.7% (for EAI), and between -0.4% and 18.8% (for RP). Varying only socio-economic parameters, changes in damages to built assets vary between -0.3% and 1.8% (for EAI), and between 0.2% and 2.3% (for RP). The authors find[13] that combining climate change and socio-economic impacts amplifies the effects, leading to a total variability range for changes in damages between 0.2% and 52.9% (for EAI), and 3.8% and 43.9% (for RP100).

To proceed, we focus on Supplementary Equation 5, i.e. we quantify the constant c that enables us to approximately scale the effects of the variation in wind speed to the effects of the total input variation.

Most of the output variability is stemming from the selection of GCM. Unfortunately, the variation in wind speed underpinning the results in (Meiler et al., 2023)[13] is not available. However, the first and total order Sobol indices are available. We observe that the first-order and total-order Sobol indices are approximately 0.7, i.e. the uncertainty on the GCM contributes to roughly 70% of the total uncertainty. We now work in the RP100 case only. We assume that the GCM uncertainty accounts for 70% of the total variability from climate change, namely 28.07%. Other climate factors would then contribute 30% of the total variability from climate change, namely 12.03%. We assume our wind speed uncertainty is only a portion of the total uncertainty across all GCMs in (Meiler et al., 2023)[13], at 1/(number of GCMs) = 1/9 = 11%. Thus, the uncertainty we computed represents only 7.7%, approximately, of the total variation induced by climate change uncertainty. Furthermore, climate change leads to only half of the total uncertainty. Thus, the sensitivity to socio-economics parameters and interaction effects leads to doubling the climate change effect. This implies that our wind speed uncertainty range represents only 4% of the total uncertainty in this calculation. In other words, the

Scenario	EAI (%)	RP250 (%)
Overall	-0.75	-3.7
SSP2-RCP6.0, 2035	-0.98	-3.6
SSP2-RCP6.0, 2040	-0.95	-3.7
SSP2-RCP6.0, 2045	-0.53	-3.6
SSP2-RCP6.0, 2050	-0.17	-3.4
SSP3-RCP2.6, 2035	-1.1	-3.7
SSP3-RCP2.6, 2040	-0.92	-3.6
SSP3-RCP2.6, 2045	-0.73	-3.4
SSP3-RCP2.6, 2050	-0.52	-3.2
SSP3-RCP4.5, 2035	-0.94	-3.8
SSP3-RCP4.5, 2040	-0.84	-3.9
SSP3-RCP4.5, 2045	-0.70	-3.9
SSP3-RCP4.5, 2050	-0.53	-4.0
SSP5-RCP4.5, 2035	-0.95	-3.8
SSP5-RCP4.5, 2040	-0.82	-3.9
SSP5-RCP4.5, 2045	-0.72	-4.0
SSP5-RCP4.5, 2050	-0.59	-4.0

Supplementary Table 5: Portfolio-level losses, equally weighted across scenarios ("Overall") and for each scenario and year combination. "Chronic + Return Period (RP) 250, asset-level" case only. First column: scenario specifications, defined by Shared Socio-Economic Pathway (SSP) - Representative Concentration Pathway (RCP) and year combination. Second column: portfolio loss, EAI (mean). Third column: portfolio loss, RP250 (VaR). Source: authors.

scaling factor between quantified uncertainty (in our model) and total uncertainty (from (Meiler et al., 2023)[13]) is 25.

After deriving c, we need to combine it with the partial derivative of the loss with respect to the wind speed, and the initial wind speed variation, to obtain the final uncertainty range (Supplementary Equation 7). If we multiply c by the partial derivative previously computed (0.25 for RP250), we obtain a scaled derivative of 6.25. This means that, for a 1% uncertainty on total inputs (i.e., including all parameters), there is a 6.25% uncertainty in output, i.e., portfolio VaR. Thus, the total uncertainty range applying Supplementary Equation 7 has a width of $\Delta Loss' \sim \Delta Impact'_m = \Delta x_{ws} c \frac{\delta Loss}{\delta x_{ws}} = 107.79\% * 25 * 0.23 = 6.20$, which, scaled to the original estimate of the VaR, leads to an upper bound for the uncertainty range given by [-3.2%, -19.85%].

Note that the results presented in this section represent only a first and approximate calculation for an upper uncertainty bound. They should be intended as an initial uncertainty analysis, while a full uncertainty analysis could be the object of later studies.

References

1. Schaeffer, R. *et al.* Energy sector vulnerability to climate change: a review. *Energy* **38,** 1–12 (2012).
2. Hong, H., Karolyi, G. A. & Scheinkman, J. A. Climate finance. *Review of Financial Studies* **33,** 1011–1023 (2020).
3. Battiston, S., Mandel, A., Monasterolo, I., Schütze, F. & Visentin, G. A climate stress-test of the financial system. *Nature Climate Change* **7,** 283–288 (Apr. 2017).
4. Eboli, F., Parrado, R. & Roson, R. Climate-change feedback on economic growth: explorations with a dynamic general equilibrium model. *Environment and Development Economics* **15,** 515–533 (2010).
5. Parrado, R. & De Cian, E. Technology spillovers embodied in international trade: intertemporal, regional and sectoral effects in a global CGE framework. *Energy Economics* **41,** 76–89 (Jan. 2014).
6. Bosello, F. *et al.* D2.7. Macroeconomic, spatially-resolved impact assessment. Deliverable of the H2020 COACCH project. 2020.
7. Delpiazzo, E. *et al.* D3.4. Macro-economic impact assessment of cascading effects of climate Change. Deliverable of the H2020 CASCADES project. 2021.
8. Aznar-Siguan, G. *et al.* CLIMADA-project/climada_python: v3.2.0. Version v3.2.0. July 2022.
9. Bresch, D. N. & Aznar-Siguan, G. CLIMADA v1.4.1: towards a globally consistent adaptation options appraisal tool. *Geoscientific Model Development* **14,** 351–363 (2021).
10. Sharpe, W. F., Alexander, G. J. & Bailey, J. V. Investments 6th (Prentice-Hall, Englewood Cliffs, 1999).
11. DiCiccio, T. J. & Efron, B. Bootstrap confidence intervals. *Statistical Science* **11,** 189–228 (1996).
12. Knutson, T. R. *et al.* Global projections of intense tropical cyclone activity for the late twenty-first century from dynamical downscaling of CMIP5/RCP4.5 scenarios. *Journal of Climate* **28,** 7203–7224 (2015).

13. Meiler, S., Ciullo, A., Kropf, C. M., Emanuel, K. & Bresch, D. N. Uncertainties and sensitivities in the quantification of future tropical cyclone risk. *Communications Earth & Environment* **4,** 371 (2023).
